# Pharmacological Basis for Use of a Novel Compound in Hyperuricemia: Anti-Hyperuricemic and Anti-Inflammatory Effects

**DOI:** 10.3389/fphar.2021.772504

**Published:** 2021-11-08

**Authors:** Lei Zhao, Yihang Li, Dahong Yao, Ran Sun, Shifang Liu, Xi Chen, Congcong Lin, Jian Huang, Jinhui Wang, Guang Li

**Affiliations:** ^1^ Department of Medicinal Chemistry and Natural Medicine Chemistry, College of Pharmacy, Harbin Medical University, Harbin, China; ^2^ Yunnan Branch, Institute of Medicinal Plant, Chinese Academy of Medical Sciences, Peking Union Medical College, Jinghong, China; ^3^ Yunnan Key Laboratory of Southern Medicinal Utilization, Jinghong, China; ^4^ School of Pharmaceutical Sciences, Shenzhen Technology University, Shenzhen, China

**Keywords:** hyperuricemia, xanthine oxidase, NF-κB, NLRP3 inflammasome, urate reabsorption transporter

## Abstract

**Background:** The prevalence of hyperuricemia is considered high worldwide. Hyperuricemia occurs due to decreased excretion of uric acid, increased synthesis of uric acid, or a combination of both mechanisms. There is growing evidence that hyperuricemia is associated with a decline of renal function.

**Purpose:** This study is aimed at investigating the effects of the novel compound on lowering the serum uric acid level and alleviating renal inflammation induced by high uric acid in hyperuricemic mice.

**Methods:** Hyperuricemic mice model was induced by potassium oxonate and used to evaluate the effects of the novel compound named FxUD. Enzyme-linked immunosorbent assay was used to detect the related biochemical markers. Hematoxylin-eosin (HE) staining was applied to observe pathological changes. The mRNA expression levels were tested by qRT-PCR. The protein levels were determined by Western blot. In parallel, human proximal renal tubular epithelial cells (HK-2) derived from normal kidney was used to further validate the anti-inflammatory effects *in vitro*.

**Results:** FxUD administration significantly decreased serum uric acid levels, restored the kidney function parameters, and improved the renal pathological injury. Meanwhile, treatment with FxUD effectively inhibited serum and liver xanthine oxidase (XOD) levels. Reversed expression alterations of renal inflammatory cytokines, urate transporter 1 (URAT1) and glucose transporter 9 (GLUT9) were observed in hyperuricemic mice. Western blot results illustrated FxUD down-regulated protein levels of inflammasome components. Further studies showed that FxUD inhibited the activation of NF-κB signaling pathway in the kidney of hyperuricemic mice. In parallel, the anti-inflammatory effect of FxUD was also confirmed in HK-2.

**Conclusion:** Our study reveals that FxUD exhibits the anti-hyperuricemic and anti-inflammatory effects through regulating hepatic XOD and renal urate reabsorption transporters, and suppressing NF-κB/NLRP3 pathway in hyperuricemia. The results provide the evidence that FxUD may be potential for the treatment of hyperuricemia with kidney inflammation.

## Introduction

Hyperuricemia is a metabolic disorder characterized by an excessively increased serum urate concentration, which may occur due to overproduction and/or insufficient intestinal excretion and/or urate underexcretion of kidney (Lu et al., 2019). Uric acid is the ultimate oxidation product of purine catabolism, which induces the formation and deposition of monosodium urate crystals and eventually leads to gout. Therefore, hyperuricemia is the main risk factor for gout (Dalbeth et al., 2018). Hyperuricemia is common and its prevalence has been increasing globally (Dehlin et al., 2020). Hyperuricemia occurs primarily in higher primates, including human, as a result of the inactivation of uricase genes during human evolution. Serum uric acid concentration is an important indicator for human health (Chen et al., 2016). When the uric acid level surpasses its solubility point of 6.8 mg/dl, hyperuricemia is believed to have developed (Terkeltaub, 2010). Alteration of serum uric acid homeostasis has been related to multiple diseases. For instance, an abnormally high serum uric acid is the root cause of gout and has been intimately associated with cardiovascular disease and renal disease (Galassi and Borghi, 2015).

Uric acid crystals formed or deposited in the kidney can induce kidney injury (Waisman et al., 1975). In the process of hyperuricemia-induced kidney damage, the deposition of uric acid crystals has an association with a common pathway that triggers kidney inflammation and injury (Herlitz et al., 2012). Innate immune pathways are also increasingly considered to play an important role in the pathogenesis of hyperuricemia (Kielstein et al., 2020), and particularly lead to activation of the NLRP3 inflammasome which contributes to the release of IL-1β and other pro-inflammatory cytokines (So and Martinon, 2017). Additionally, increased cellular urate, oxidative stress induced directly or indirectly by xanthine oxidase can be related to inflammasomes (Isaka et al., 2016). The processing of uric acid in the kidney mainly encompasses glomerular filtration, tubular reabsorption, tubular secretion, and reabsorption after secretion (Mendez Landa, 2018). Uric acid transporters are necessary for the kidney to handle uric acid, and can be grossly classified into reabsorption-related and secretion-related proteins (Su et al., 2020). Reabsorption-related proteins primarily consist of urate anion transporter 1 (URAT1), glucose transporter 9 (GLUT9), and organic anion transporter 4 (OAT4) (Hagos et al., 2007). For example, the clearly defined function of URAT1 is to promote reabsorption of uric acid at the apical membrane of proximal tubule epithelial cells (TECs) (Enomoto et al., 2002). GLUT9 functions as a transporter that reabsorbs both uric acid and glucose into tubular cells (Chiba et al., 2020). Accordingly, the regulation of cellular uric acid transporters would be able to result in alteration in urate excretion in the kidney (Nakayama et al., 2011). The mechanisms and pathways through which elevated uric acid could impact renal function and eventually lead to kidney damage, have been investigated previously.

Not all patients with gout are able to tolerate allopurinol therapy, and febuxostat still has certain side effects during clinical application. Therefore, the discovery of a promising treatment option for hyperuricemia and gout is urgently needed, and it is of major importance to search for new drugs to ameliorate hyperuricemia. Although the symptoms of kidney injury caused by hyperuricemia have long been known, the underlying molecular mechanisms have not been fully understood. Inflammation is an important physiological response to defense against noxious stimuli, such as pressure on tissues, infection or injury (Rock et al., 2013). Uric acid has been well-known for a long time, but there are few new experimental researches and discoveries around it (Medzhitov, 2008). In our study, we synthesized a novel compound named FxUD and its specific role in those pathological states accompanied by an underlying inflammatory process deserved further investigations. To be specific, we aimed to investigate the protective role of FxUD on high uric acid-induced renal injury, and in the meantime, understanding the specific underlying molecular mechanism was another important task.

## Materials and Methods

### Reagents and Kits

**FIGURE 1:**
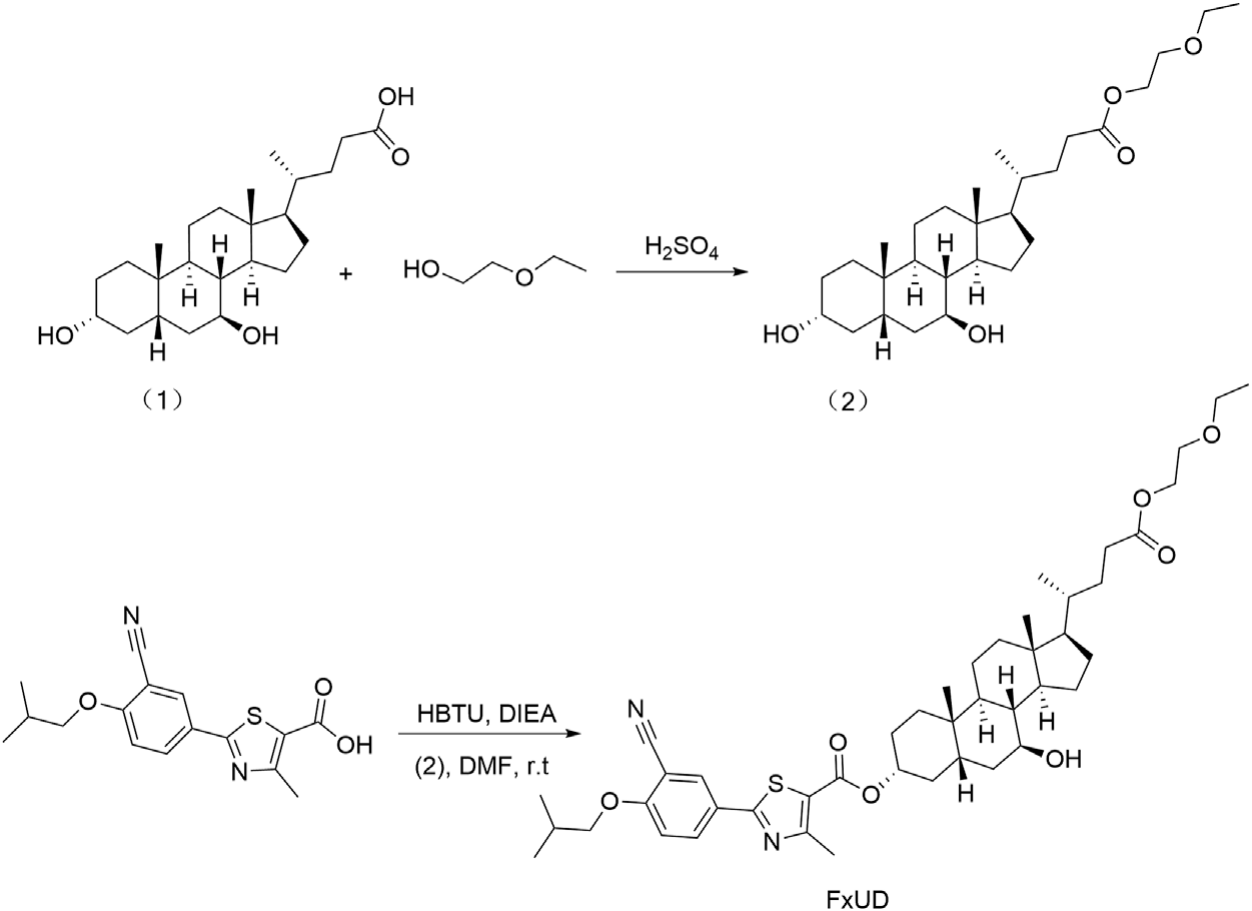
The synthetic route of the novel compound named FxUD.

FxUD is a pale yellow-white crystalline powder. Its chemical structure and synthetic routes are shown in Figure 1. Unless otherwise noted, reagents used in the experiment were purchased from Aladdin and used without further purification. The uric acid assay kit was purchased from Elabscience. Blood urea nitrogen (BUN) assay kit and creatinine (Cr) assay kit were purchased from Jiangsu Meibiao Biotechnology (Mbbiology). Xanthine oxidase (XOD) detection kit was purchased from Jianglai Biotechnology. LDH assay kit was purchased from Nanjing Jian Cheng Bioengineering Institute. The enhanced chemiluminescence detection kit was purchased from Kangwei Century. The bicinchoninic acid (BCA) and Cell Counting Kit-8 (CCK-8) assay kit were purchased from Beyotime Biotechnology. The hematoxylin-eosin (HE) staining kit was purchased from Solarbio Life Sciences.

### Animals

Healthy ICR male mice weighing 20–25 g of SPF grade were used. They were allowed to acclimatize to their living environment for at least 7 days before the formal experiments. The animals were housed in air-conditioned room at 25 ± 2°C with relative humidity (60 ± 5%) in a regular 12 h light/dark cycle with free access to a standard mouse chow and water during the experimental period. All procedures were in line with the Experimental Animal Ethics Committee of Harbin Medical University.

### Animal Treatment, Drug Administration and Samples Collection

According to the previous studies, hyperuricemia was induced in mice by uricase inhibitor potassium oxonate (PO) (Wang et al., 2016; Wang et al., 2018). In briefly, the mice were allocated into six groups (*n* = 10/group) randomly. An hour after the food was withdrawn, PO (250 mg/kg/day) was administered by gavage for seven consecutive days. FxUD (6, 12, 24 mg/kg/day) and febuxostat (FEB) (5 mg/kg/day), dissolved in 0.5% carboxymethyl cellulose sodium (CMC-Na), were administered 1 h after PO administration, respectively. Blood samples were obtained by extracting the eyeball after final administration on the 7 th day, allowed to clot for approximately 1 h at room temperature, and then centrifuged at 10,000×*g* at 4°C for 5 min (Qin et al., 2018) in order to collect the serum. Following this, mice were euthanized by decapitation. Meanwhile, liver tissues were taken and kidneys were dissected on an ice plate quickly and some parts were immediately fixed for HE staining, other parts were conserved at −80°C for further qRT-PCR and Western blot analysis.

### Biochemical Assays

Serum and hepatic XOD levels were detected by XOD detection kits according to the manufacturer’s instructions. Serum Cr, BUN, ALT, AST and LDH levels were determined in accordance with the manufacturer’s protocols.

### RNA Isolation and Quantitative Reverse Transcription-PCR Analysis

Total RNA was extracted using the using Foregene RNA isolation kit (Foregene Co. Ltd., China) according to the manufacturer’s instructions. Reverse transcription was performed using the TOYOBO ReverTra Ace^®^ qPCR RT Master Mix (Osaka, Japan) after RNA quantification. Real-time PCR was performed using the PowerUP™ SYBR™ Green Master Mix (Thermo Fisher Scientific). The comparative Ct (2^−ΔΔCt^) method was used to determine the relative mRNA expression, normalized to GAPDH. The used primers were listed as below: XOD, 5ʹ-TCA​GAA​GCC​AAG​AAG​GTG-3ʹ and 5ʹ-ATG​TTC​TGG​GGT​GTC​AGC-3ʹ; IL-1β, 5′-CTC​ACA​AGC​AGA​GCA​CAA​GC-3′ and 5′-CAG​TCC​AGC​CCA​TAC​TTT​AGG-3′; TNF-α, 5′-CCT​GGA​GGA​GAA​GAG​GAA​AGA​GA-3ʹ and 5′-TTG​AGG​ACC​TCT​GTG​TAT​TTG​TCA​A-3ʹ; IL-6, 5′-CCA​TCC​AGT​TGC​CTT​CTT​GG-3ʹ and 5′-TGC​AAG​TGC​ATC​ATC​GTT​GT-3ʹ; MCP-1, 5ʹ-TAA​AAA​CCT​GGA​TCG​GAA​CCA​AA-3ʹ and 5ʹ-GCA​TTA​GCT​TCA​GAT​TTA​CGG​GT-3ʹ; URAT1, 5ʹ-AGC​TCT​TGG​ACC​CCA​ATG​C-3ʹ and 5ʹ-CTT​CAG​AGC​GTG​AGA​GTC​ACA​CA-3ʹ; GLUT9, 5ʹ-TCT​CAG​TTG​CTT​GGG​AGC​AG-3ʹ and 5ʹ-AGC​TAA​AGC​AAG​CTC​CCT​GG-3ʹ; GAPDH, 5ʹ-GCT​GAG​TAT​GTG​GAG​T-3ʹ and 5ʹ-GTT​CAC​ACC​CAT​CAC​AA AC-3ʹ.

### Western Blot Analysis

Western blot analysis was performed as previous study with some modifications (Faqihi et al., 2020). In briefly, tissues or cells protein extracts were prepared by using radioimmunoprecipitation (RIPA) lysis buffer (Beyotime) and then centrifuged at 12,000×*g* at 4°C for 20 min. The protein concentration was determined by BCA protein assay kit. Nuclear and cytoplasmic protein extraction was performed using a Nuclear and Cytoplasmic Protein Extraction Kit (Beyotime). After electrophoresis, separated proteins were transferred from the SDS-polyacrylamide gels to a nitrocellulose membrane. Then, the membranes were blocked with 5% skim milk for 2 h at room temperature and incubated with the indicated primary antibodies at 4°C overnight for IL-1β, TNF-α, IL-6, MCP-1, URAT1, GLUT9, NLRP3, ASC, c-caspase-1, NF-κB, p-NF-κB, IκBα, p-IκBα, β-actin and Histone H3. Next day, the membranes were washed three times, and then they were incubated with secondary antibodies corresponding to the respective species of primary antibodies for 1 h at room temperature. The bands were scanned by a gel imaging system using enhanced chemiluminescence detection kit. The grayscale values were analyzed by Image J software.

### Histopathological Examination

Tissues were fixed with 4% paraformaldehyde for 24 h, and then embedded in paraffin and sectioned transversely at 4 µm for HE staining. The stained sections were visualized under light microscopy at 200× magnifications.

### Cell Culture and Treatments

Human renal proximal tubule epithelial cell line (HK-2) was maintained in RPMI-1640 medium with 10% fetal bovine serum, 100 U/ml penicillin and 100 μg/ml streptomycin (Hou et al., 2019) in a humidified incubator under an atmosphere of 95% air and 5% CO_2_ for further study. The medium was changed every other day. After 12 h starvation in serum-free cell culture medium, HK-2 cells were subsequently incubated with 0.1% DMSO (control group), uric acid (4 mg/dl) and uric acid combined with 200 µM FxUD for 24 h, respectively. In addition, to further explore the role on pathway, HK-2 cells were pre-treated with NF-κB inhibitor SN50 (20 µM) or NLRP3 inhibitor MCC950 (10 µM) (MCE) for 1 h and then were stimulated with uric acid for 24 h.

### Cell Viability Analysis

Cell viability was measured by CCK-8 assay kit. HK-2 cells were seeded into 96-well plates at a density of 2 × 10^5^ cells/well and allowed to adhere overnight. After treatment with various concentrations of chemicals for 24 and 48 h, or treated with various concentration of uric acid for 24 h, 10 µL CCK-8 regent was added to each well of 96-well plates, and the plate was incubated at 37°C for 2–4 h following the manufacturer’s protocols. Absorbance was measured at 450 nm using a SpectraMax M3 microplate reader.

### Statistical Analysis

Statistical analysis was performed with the GraphPad Prism 8.0 software package. All data were presented as the mean ± standard deviation (SD). Comparison among and between groups was analyzed with one-way analysis of variance (ANOVA) and Student’s t-test, respectively. And statistical significance was concluded at *p < 0.05.

## Results

### FxUD Decreases Serum Uric Acid Levels and Ameliorates Renal Function in Hyperuricemic Mice

**FIGURE 2:**
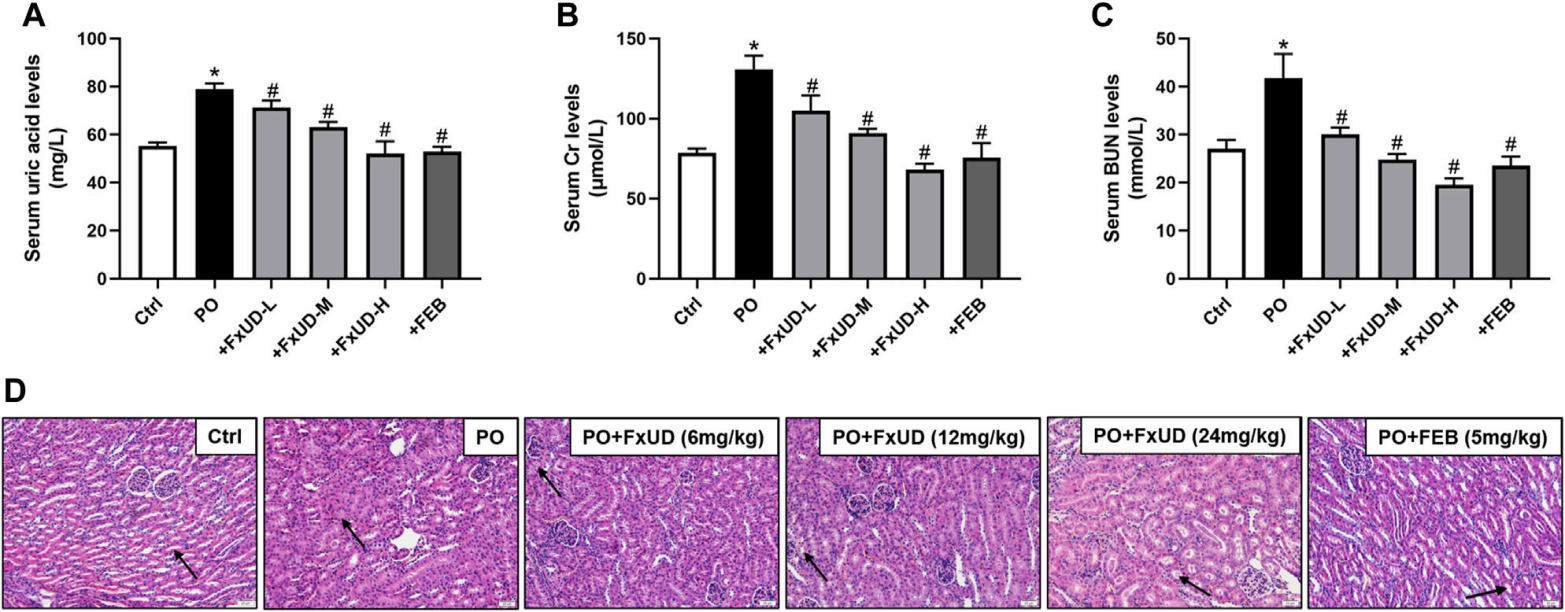
FxUD reduces serum uric acid levels and restores renal function in hyperuricemic mice. **(A)** Uric acid, **(B)** Cr and **(C)** BUN levels in serum from the mice (*n* = 8). **(D)** The representative pictures of renal histopathology in each group (*n* = 4). Kidneys in control group maintained a normal renal tubular structure (arrow) with no evidence of inflammation, kidneys in PO group exhibited inconspicuous boundaries between adjacent tubule cells and swelling, while regulated tubule cells and the cytoplasm were relatively clear in kidneys of high-dose FxUD or FEB treatment group. Magnification 200×, scale bar 20 µm. Values are expressed as the means ± SD. *p < 0.05 vs. Ctrl, ^#^ *p* < 0.05 vs. PO. Ctrl, control; PO, potassium oxonate; FEB, febuxostat; Cr, creatinine; BUN, urea nitrogen.

We firstly assessed uric acid levels in the serum of mice. As shown in Figure 2A, after 7 days of potassium oxonate (PO) administration, the PO group exhibited higher serum uric acid levels compared to control group, indicating that hyperuricemia was successfully established. Uric acid levels in the serum were significantly reduced in the FxUD and febuxostat (FEB) group in comparison to the PO group, and the inhibitory effect of FxUD appeared to be dose dependent. Uric acid is mainly excreted by the kidney, and a small amount is excreted by the gastrointestinal tract. Insufficient excretion of uric acid can cause kidney damage (Pilemann-Lyberg et al., 2019). As the creatinine (Cr) and blood urea nitrogen (BUN) levels in serum are effective indicators of renal function, the levels of Cr and BUN in the serum of each group were detected by detection kit. As expected, in comparison with the control group, Cr (Figure 2B) and BUN (Figure 2C) levels in the PO group were significantly increased. After treatment with FxUD and FEB, the serum Cr and BUN levels were significantly decreased to approximately the normal values. The inflammatory state and some changes in tissue structure were observed as obvious pathological features of hyperuricemia in clinical trials (Gupta and Singh, 2019). Histopathological examination for renal tissues of the mice was shown in Figure 2D. Kidney sections isolated from control group mice maintained a normal kidney structure, without obvious inflammatory responses. The normal appearance of renal glomerulus, tubules and interstitium stayed with a compact arrangement of cells was seen. However, the kidneys in PO group mice displayed histological alterations consist of inconspicuous boundaries between adjacent proximal tubule cells and swelling. The kidneys of mice treated with high-dose FxUD and FEB showed restoration of normal tubular histology to some extent. The above results indicated that FxUD could ameliorate the kidney damage induced by hyperuricemia.

### FxUD Exerts Ameliorative Effects on Changes of Liver Biomarkers and Hepatic XOD in Hyperuricemic Mice

**FIGURE 3:**
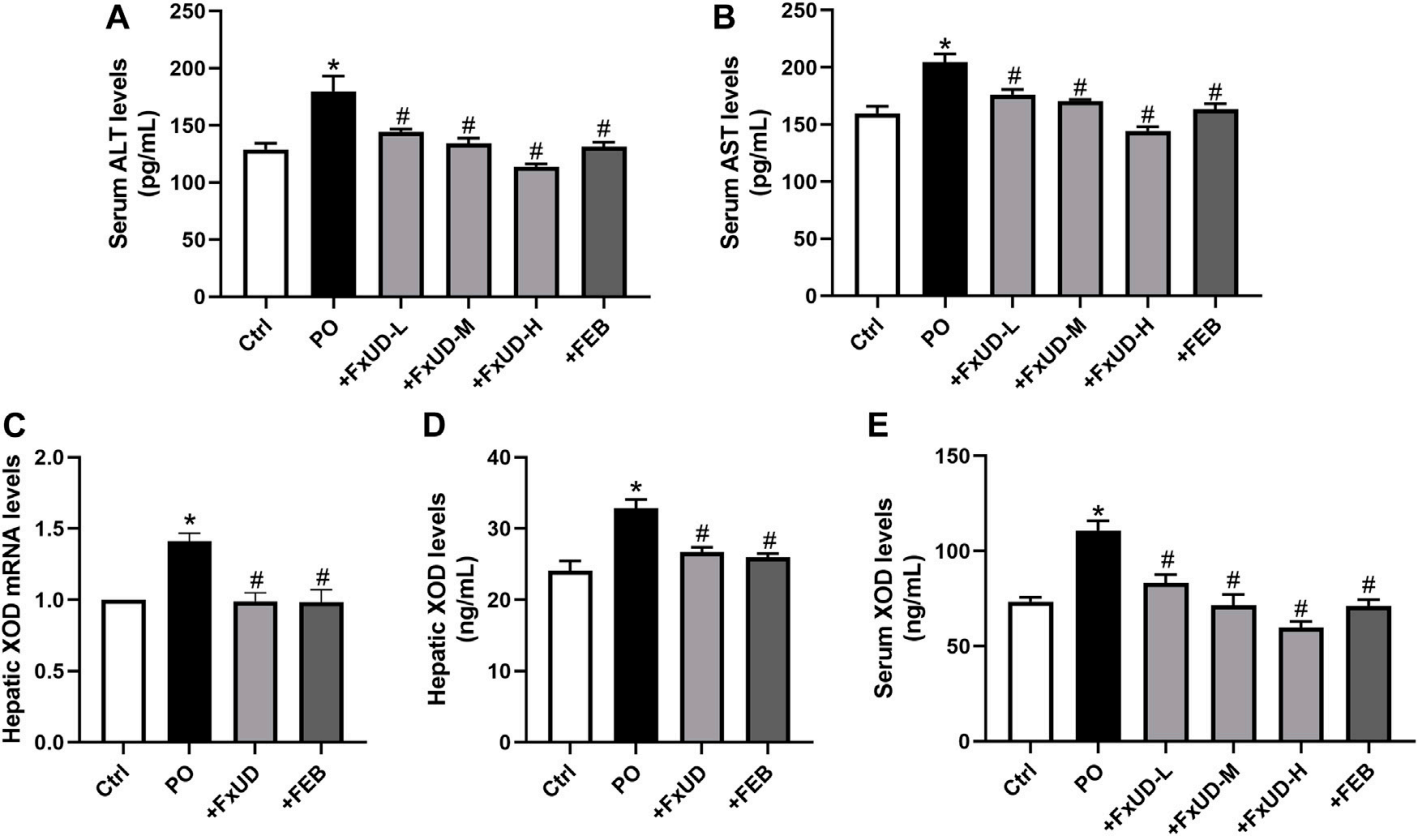
Ameliorative effects of FxUD on changes of liver biomarkers and hepatic XOD in hyperuricemic mice. **(A)** ALT and **(B)** AST levels in mice serum as indicators of liver function (*n* = 8). **(C)** XOD mRNA expression (*n* = 5). **(D)** XOD protein levels in the liver were detected by ELISA kit (*n* = 8). **(E)** XOD in serum of mice (*n* = 8). Values are expressed as the means ± SD. *p < 0.05 vs. Ctrl, ^#^ *p* < 0.05 vs. PO. ALT, alanine aminotransferase; AST, aspartate aminotransferase; XOD, xanthine oxidase.

Uric acid is the end product of the catabolism of purine compounds in the liver. We tested alanine aminotransferase (ALT) and aspartate aminotransferase (AST) levels in mice serum as indicators for liver function (Figures 3A,B). Administration of PO increased the levels of serum ALT and AST in hyperuricemic mice compared to control mice. FxUD treated hyperuricemic mice exhibited decreased altered parameters. In the process of purine metabolism, xanthine oxidoreductase is a key enzyme that catalyzes the oxidation of hypoxanthine and xanthine to form the final product uric acid. Therefore, xanthine oxidoreductase is currently considered to be the most promising target for preventing the accumulation of uric acid in the treatment of hyperuricemia (Smelcerovic et al., 2017). As shown in Figures 3C,D, qRT-PCR and ELISA were performed to measure xanthine oxidase (XOD) mRNA and protein levels, hyperuricemic mice showed up-regulation of XOD mRNA and protein expression in the liver, however, FxUD remarkably down-regulated XOD mRNA and protein expression of hyperuricemic mice. Consistent changes were observed at both the mRNA and protein levels in FEB group. Simultaneously, XOD in serum (Figure 3E) was increased in hyperuricemic mice and was normalized significantly by FxUD. Thus, the results demonstrated a potential mechanism of uric acid-lowering property of FxUD partly was the inhibition of hepatic XOD in hyperuricemic mice.

### FxUD Reduces the Expression of Inflammatory Cytokines in the Kidneys of Hyperuricemic Mice

**FIGURE 4:**
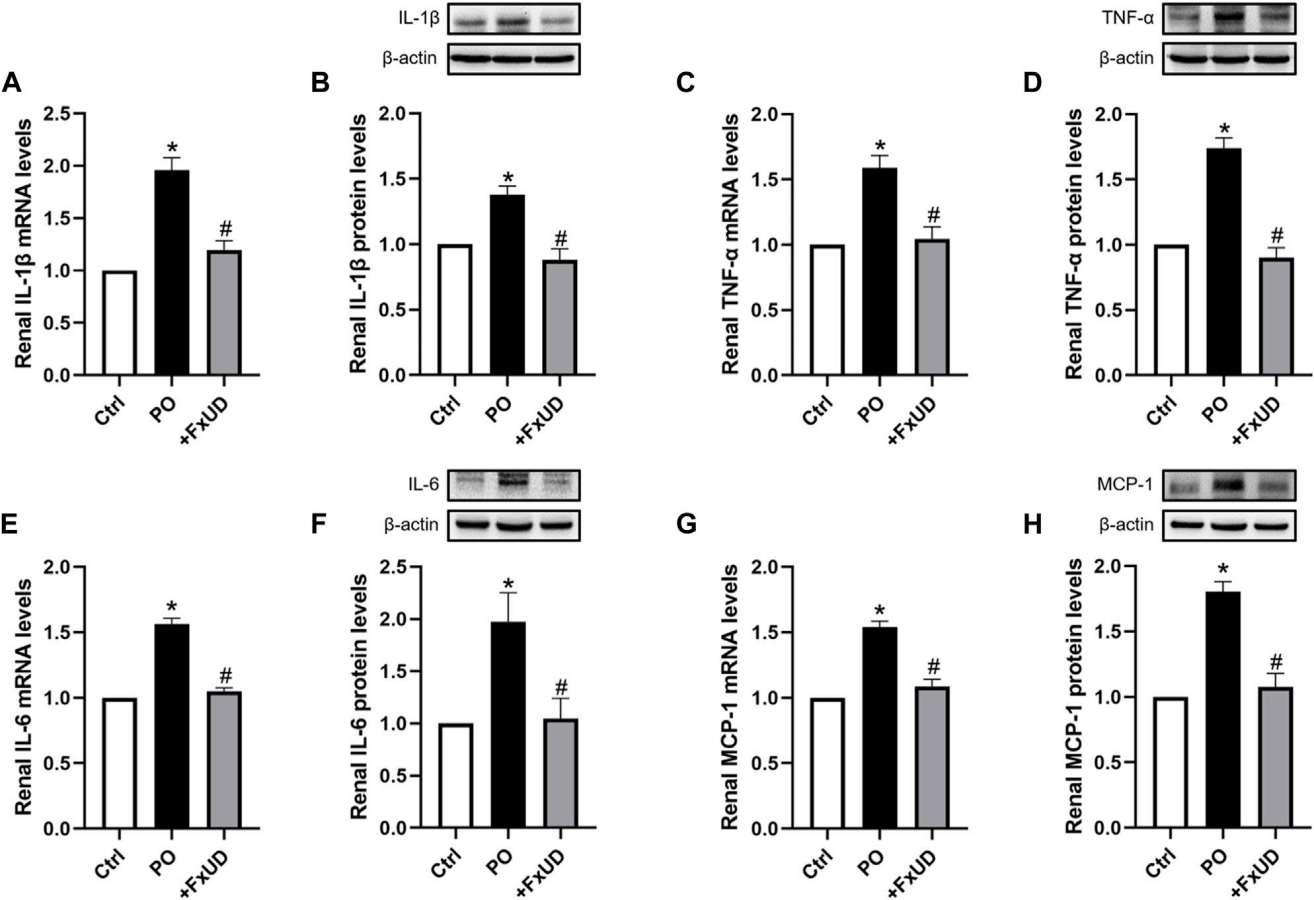
FxUD decreases the expression of renal inflammatory cytokines in hyperuricemic mice. **(A)** qRT-PCR assay and **(B)** Western blot analysis of IL-1β. **(C)** qRT-PCR and **(D)** Western blot results of TNF-α. **(E)** qRT-PCR and **(F)** Western blot results of IL-6. **(G)** MCP-1 mRNA and **(H)** protein expression in the kidney. Values are expressed as the means ± SD (*n* = 4–5). *p < 0.05 vs. Ctrl, ^#^ *p* < 0.05 vs. PO.

Inflammation can cause persistent malfunction of tissue (Dkhil et al., 2018). Thus, qRT-PCR and Western blot analysis of inflammatory factors such as cytokines IL-1β (interleukin-1β), TNF-α (tumor necrosis factor-α) and IL-6 (interleukin-6) in kidney were performed to explore the possible mechanisms. As shown in Figure 4, potassium oxonate significantly increased the mRNA levels of renal inflammatory cytokines including IL-1β, TNF-α, IL-6, and MCP-1 (monocyte chemoattractant protein-1) in hyperuricemic mice. Surprisingly, after treatment with a high dosage of FxUD, the mRNA levels of the above four inflammatory cytokines were all significantly decreased. The protein results were consistent with those of mRNA expression. These results illustrated that FxUD could suppress the expression of renal inflammatory cytokines to protect against the kidney damage induced by PO, and we therefore hypothesized that FxUD might inhibit the activation of NF-κB p65. Of course, further experiments would be needed to validate this hypothesis.

### Effects of FxUD on Uric Acid Reabsorption Transporters in the Kidneys of Hyperuricemic Mice

**FIGURE 5:**
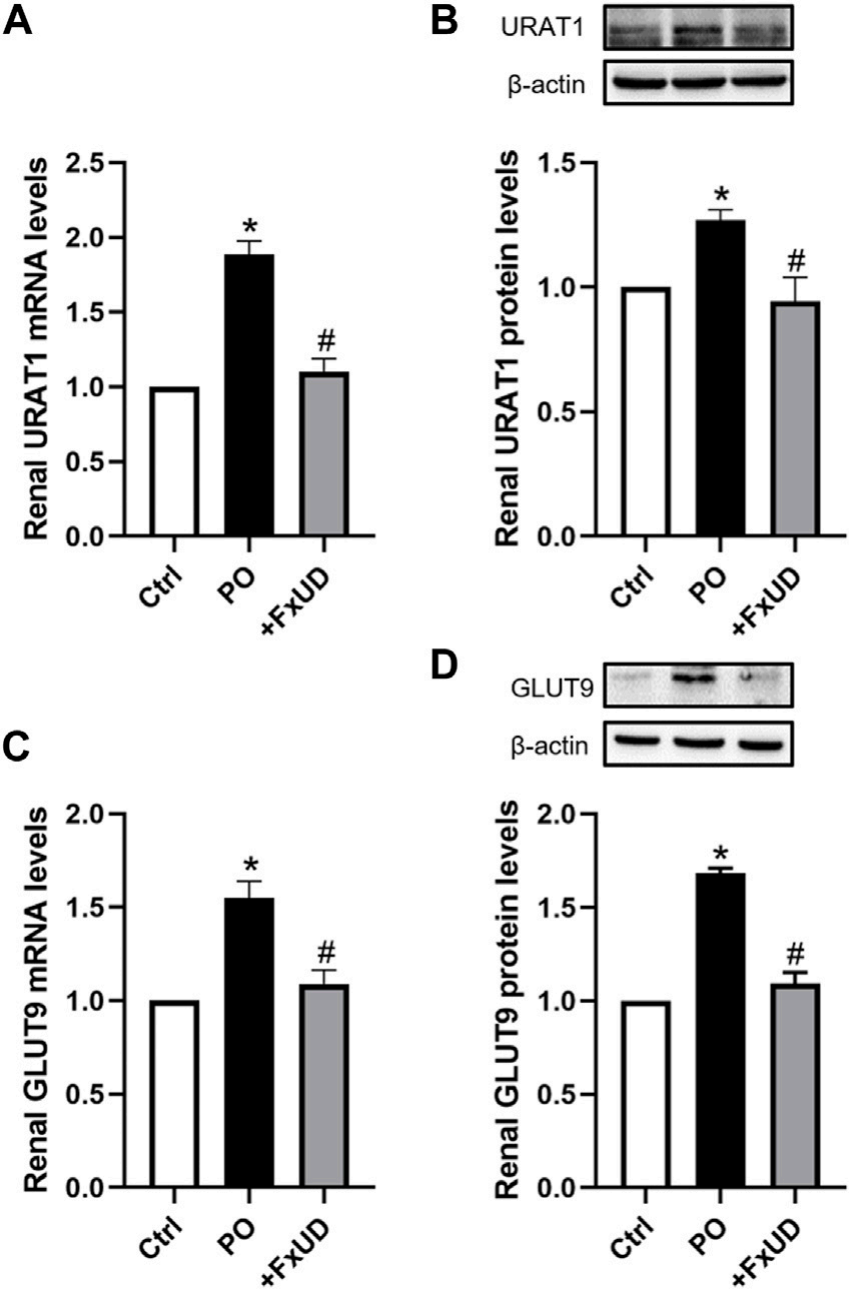
FxUD reduces renal reabsorption of uric acid in hyperuricemic mice. **(A)** mRNA and **(B)** protein levels of URAT1 in the kidney. **(C)** mRNA and **(D)** protein levels of GLUT9 in the kidney of mice. Values are expressed as the means ± SD (*n* = 4–5). *p < 0.05 vs. Ctrl, ^#^ *p* < 0.05 vs. PO.

Under-excretion of uric acid has been implicated to lead to hyperuricemia (Liote, 2003). Therefore, the renal urate transporter has become a significant physiologic target for drugs to treat hyperuricemia. Human urate transporter 1 (hURAT1) is located on the brush-border membrane of proximal tubules in the kidney. As the homolog of hURAT1, mURAT1, which has the same tissue distribution as hURAT1, participates in renal urate reabsorption and plays an important role in regulating serum uric acid alteration (Takiue et al., 2011). And mGLUT9 in mouse encoded by SLC2A9 is considered to be expressed in the apical and basolateral membrane of distal convoluted tubules and could be associated with renal urate reabsorption (Vitart et al., 2008). Therefore, we investigated whether FxUD affected renal urate transport-related proteins in PO-induced hyperuricemic mice. The changes of renal urate transporters including URAT1 and GLUT9 were analyzed (Figure 5). Our results showed that compared to the control group, the mRNA and protein expression levels of URAT1 and GLUT9 were significantly increased in hyperuricemic mice, and FxUD resulted in a significant decrease in both mRNA and protein levels of URAT1 and GLUT9 compared to PO group. The results suggested that FxUD could be a useful alternative for prevention or treatment on hyperuricemia via reducing renal reabsorption of uric acid.

### FxUD Inhibits the Activation of NLRP3 Inflammasome in the Kidneys of Hyperuricemic Mice

**FIGURE 6:**
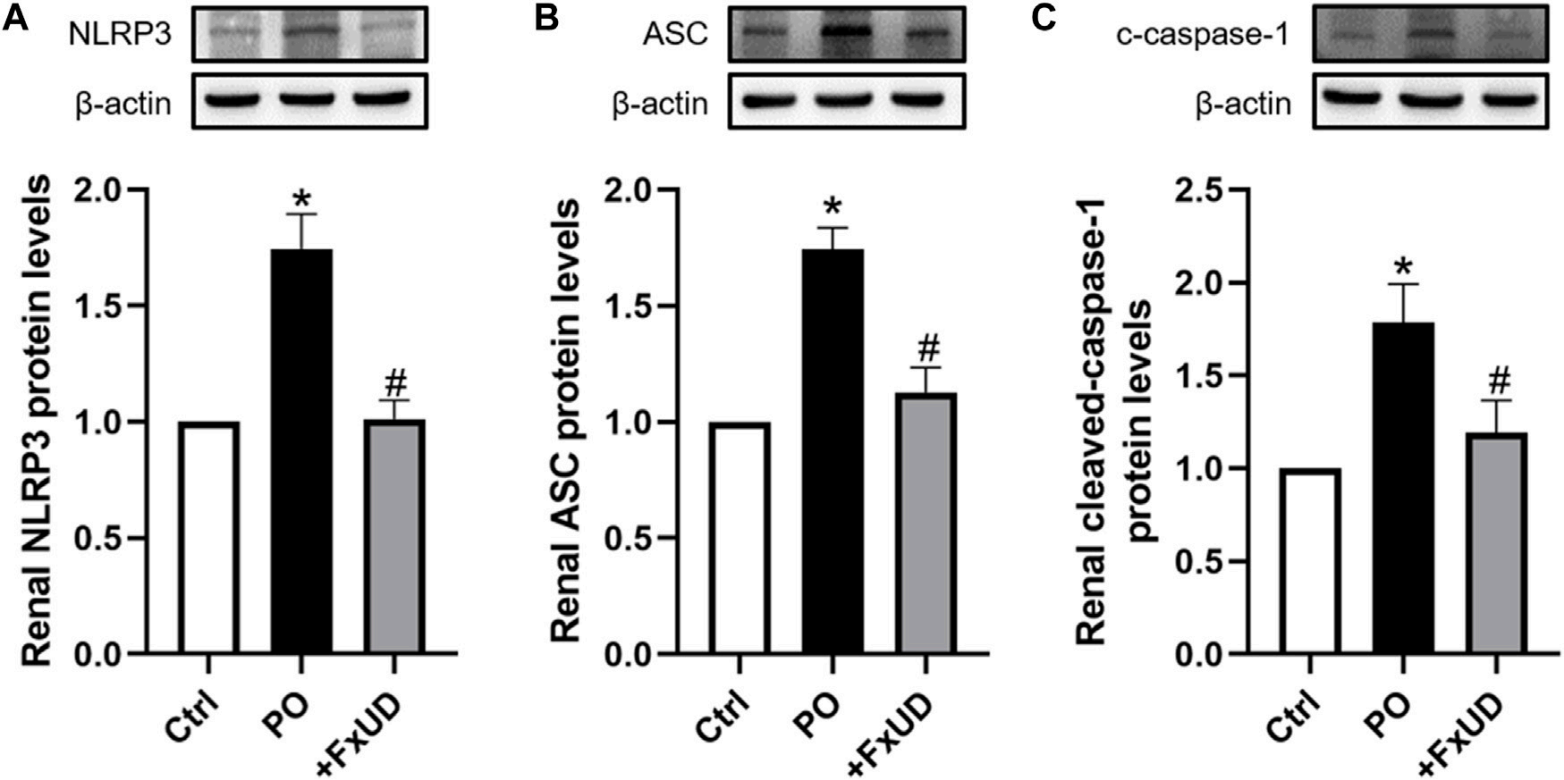
FxUD inhibits the activation of NLRP3 inflammasome in the kidney of mice with hyperuricemia. Graphic representations of the ratios **(A)** NLRP3/β-actin, **(B)** ASC/β-actin and **(C)** c-caspase-1/β-actin. β-actin was used as loading control. Values are expressed as the means ± SD (*n* = 5). *p < 0.05 vs. Ctrl, ^#^ *p* < 0.05 vs. PO.

NLRP3 (NOD-like receptor family pyrin domain containing 3) inflammasome, suggesting inflammation may be present, plays a vital role in the pathogenesis of kidney inflammation (Chen et al., 2019). The soluble urate and urate crystals have been proved to promote activation of NLRP3 inflammasome, and then trigger congenital immune against danger signals (Braga et al., 2017). In our results described above, FxUD could reduce the expression levels of inflammatory cytokine IL-1β in mice with hyperuricemia, which is downstream product of NLRP3 inflammasome pathway (Ruiz et al., 2017). On this basis, we surveyed the effects of FxUD on regulating the protein expressions of NLRP3 inflammasome signaling pathway (Figure 6). We found that the protein expression levels of NLRP3 and its downstream signaling molecules were significantly increased in the kidney tissues after treatment with PO when compared to the control group, whereas FxUD reduced these inflammatory factors in kidneys of mice with hyperuricemia suggesting that FxUD could reduce kidney inflammation through inhibiting the activation of NLRP3 inflammasome.

### FxUD Attenuates Inflammation by Suppressing NF-κB Signaling Pathway in the Kidneys of Hyperuricemic Mice

**FIGURE 7:**
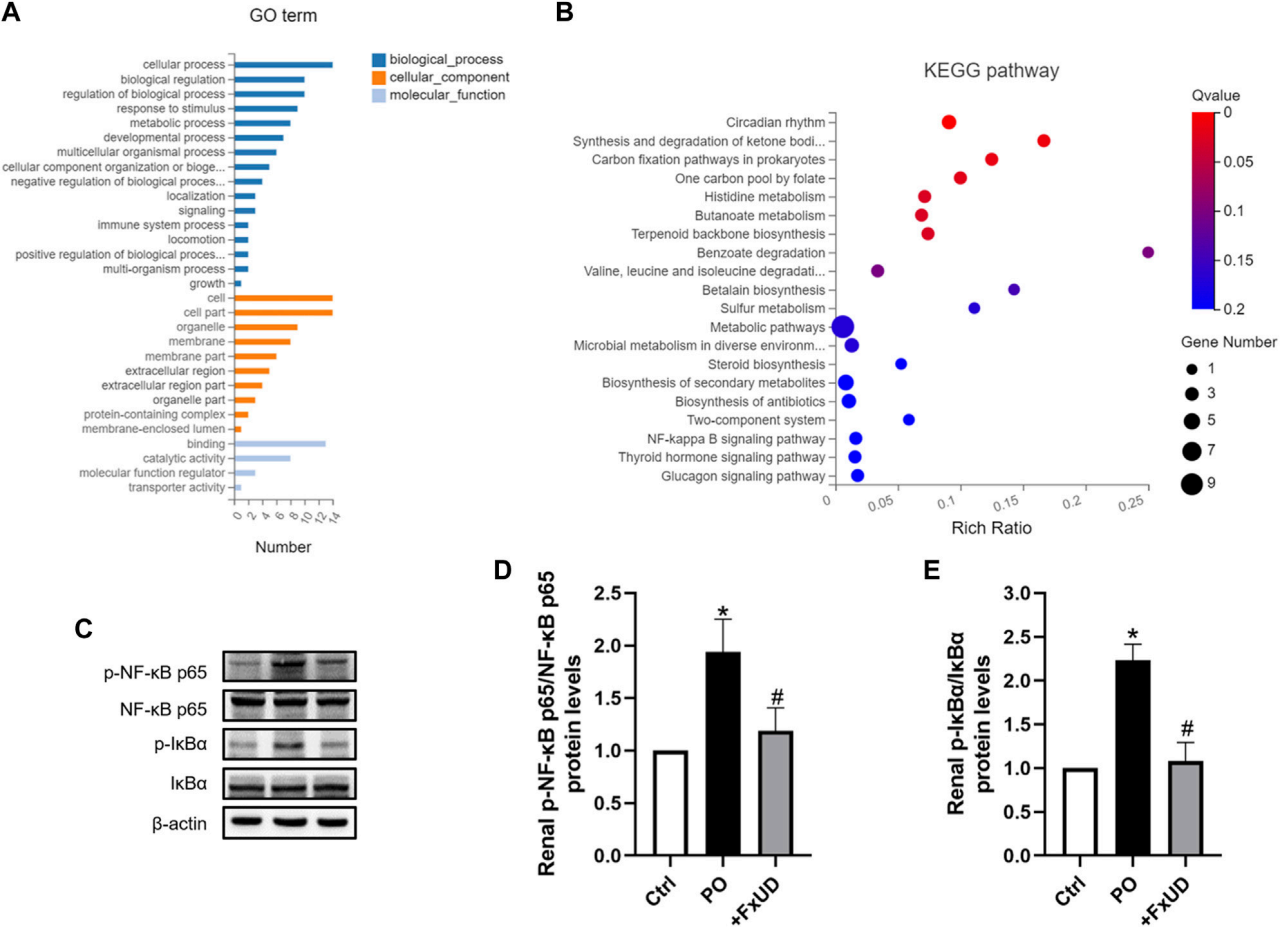
FxUD attenuates uric acid induced inflammation by suppressing NF-κB signaling pathway in the kidney of mice with hyperuricemia. **(A)** Gene ontology analysis. **(B)** The scatter plot for KEGG enrichment results. **(C)** Representative Western blot bands were shown. **(D)** Graphic presentation of relative p-NF-κB p65 and normalized to NF-κB p65 (*n* = 5). **(E)** Graphic presentation of relative p-IκBα and normalized to IκBα (*n* = 5). Values are expressed as the means ± SD. *p < 0.05 vs. Ctrl, ^#^ *p* < 0.05 vs. PO.

Once xanthine oxidase produces too much uric acid, which exceeds the excretion capacity of the kidney, the uric acid that cannot be promptly excreted will deposit in the kidney, directly leading to renal injury (Zhang et al., 2019). Uric acid crystals deposited in the kidney will induce the release of inflammatory cytokines via regulating the nuclear factor-κB (NF-κB) signaling pathway, thereby exacerbate renal injury (Li et al., 2014). To explore the molecular basis of FxUD on alleviating inflammation, 9 transcriptomes including 3 controls, 3 models, and 3 treatments, respectively, were sequenced using RNA-Seq technology. Gene ontology (GO) terms were depicted in Figure 7A. The analysis showed enrichment in pathways encompassing metabolism, innate immunity, transporter, and protein binding, as well as pathways that may indicate underlying mechanism in uric acid-induced inflammatory status. Accordingly, pathway enrichment analysis of differentially genes expressions regulated by FxUD treatment compared with the model group based on the KEGG database was performed, and a bubble plot for KEGG enrichment results was generated (Figure 7B). As expected, we found that NF-κB pathway signaling was involved in the KEGG enrichment results between the model and treat group. This suggested that NF-κB was involved in general inflammatory processes. In order to clarify the intracellular signal transduction associated with uric acid-induced inflammation, we intended to verify the targets revealed by transcriptomic analysis. The involvement of NF-κB in the priming effects of uric acid was evaluated by Western blot (Figures 7C–E). The level of phosphorylated NF-κB p65 was increased by PO treatment, and this effect was reversed by FxUD. Moreover, the phosphorylation of IκBα induced by PO was reduced by the treatment with FxUD. Together, these data suggested that FxUD might exert the inhibitory effect by suppressing the activation of NF-κB signaling pathway.

### Effects of FxUD on Inflammatory Cytokines and Uric Acid Reabsorption Transporters in HK-2 Cells

**FIGURE 8:**
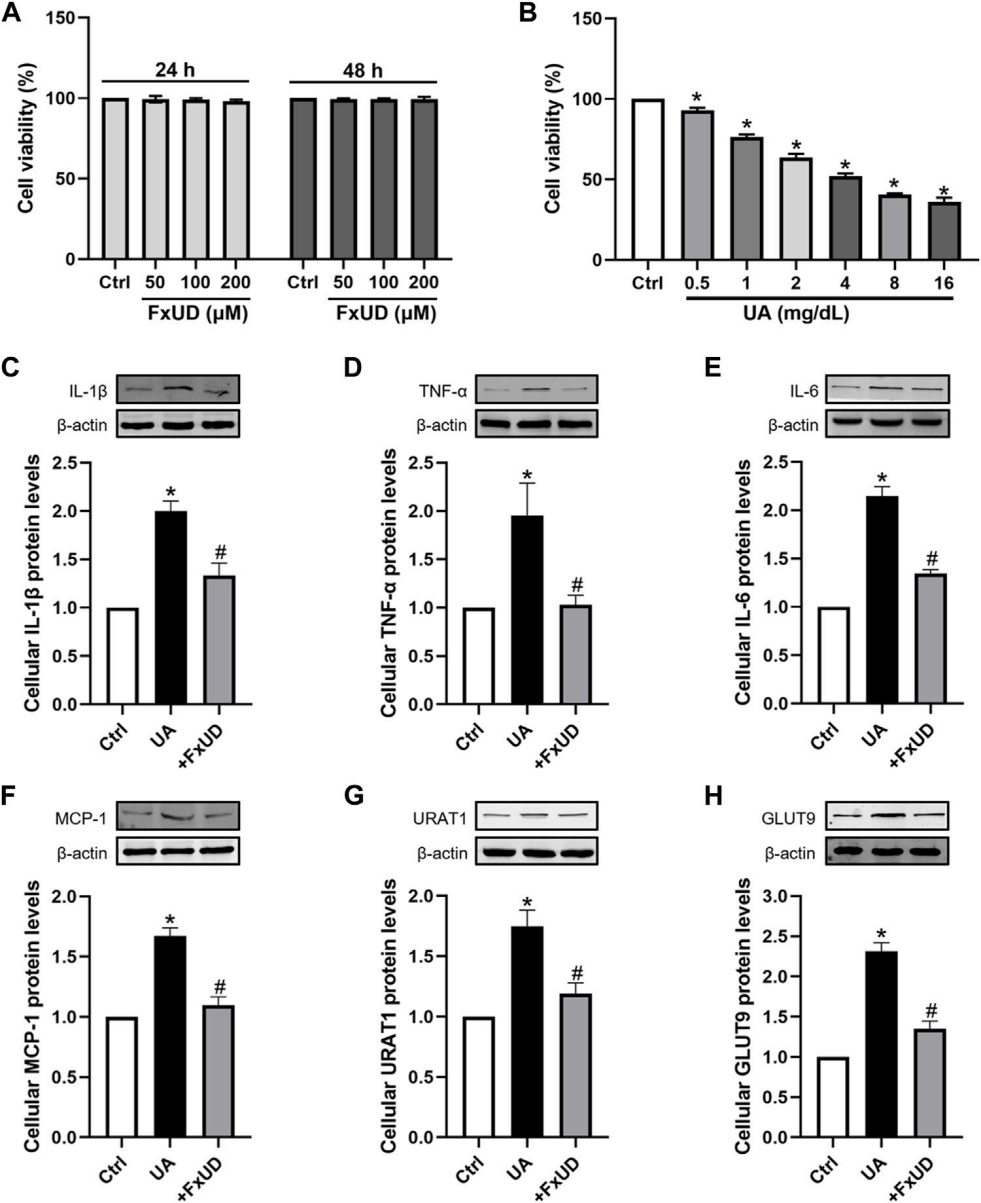
FxUD regulates the expression of inflammatory cytokines and uric acid reabsorption transporters in uric acid-exposed HK-2 cells. **(A)** Changes in the viability of HK-2 cells following 24 or 48 h of treatment with chemicals at different concentrations, measured using CCK-8 assay (*n* = 3). **(B)** Cell viability after treated with various concentrations of UA for 24 h, and the following studies were performed (*n* = 4). **(C–H)** Representative Western blot bands and protein levels of IL-1β, TNF-α, IL-6, MCP-1, URAT1 and GLUT9 in HK-2 cells, respectively (*n* = 4). Values are expressed as the means ± SD. *p < 0.05 vs. Ctrl, ^#^ *p* < 0.05 vs. UA. UA, uric acid.

A proximal tubular cell line derived from normal kidney, HK-2, was used as an *in vitro* cell model to further study the anti-inflammatory effects of FxUD. Here, we attempted to identify whether FxUD could influence cell survival rate. The effects of FxUD on cell viability were analyzed by CCK-8 assay. HK-2 cells were incubated with FxUD of various concentrations as indicated for 24 or 48 h, respectively. And the presence of FxUD had no obvious effect on cell survival (Figure 8A). Then followed, various concentrations of uric acid (UA) were incubated to HK-2 cells for 24 h. And CCK-8 analysis illustrated that uric acid dose-dependently reduced the cell viability (Figure 8B). The subsequent parts were focused on further confirming our hypothesis that FxUD could relieve renal dysfunction through inflammation inhibition and regulating urate transport-related proteins *in vitro*. As shown in Figures 8C–H. In consistent with the results from animal experiments, uric acid exposure led to higher expression of inflammatory cytokines and uric acid reabsorption transporters compared to the control group. However, FxUD administration could down-regulate those proteins expressed levels in cells. The data above indicated that FxUD could suppress inflammation-associated proteins and urate reabsorption-related proteins expressions in UA-treated HK-2 cells *in vitro*.

### Effects of FxUD on NLRP3 Inflammasome and NF-κB Signaling Pathway in HK-2 Cells

**FIGURE 9:**
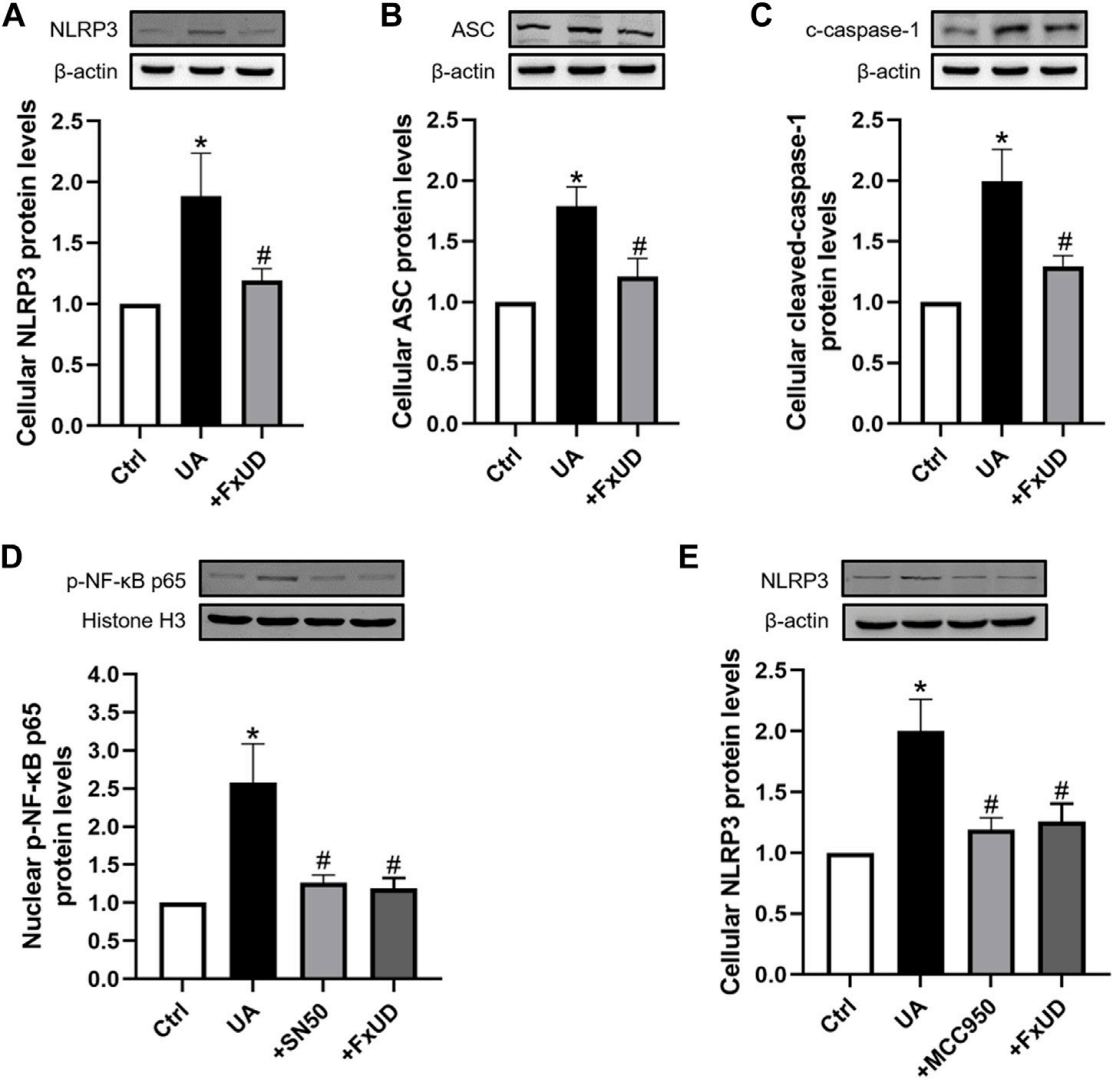
FxUD inhibits the activation of NLRP3 inflammasome and NF-κB signaling pathway in uric acid-exposed HK-2 cells. **(A)** NLRP3, **(B)** ASC, **(C)** c-caspase-1 protein levels in HK-2 cells were measured using Western blot analysis. The protein levels of **(D)** p-NF-κB and **(E)** NLRP3 in HK-2 cells exposed to UA after treatment with SN50 or MCC950. Values are expressed as the means ± SD (*n* = 4). *p < 0.05 vs. Ctrl, ^#^ *p* < 0.05 vs. UA.

As shown in Figures 9A–C, to further reveal the specific role of FxUD in NLRP3 pathway, NLRP3, ASC, cleaved-caspase-1 proteins expressions induced by uric acid treatment were also examined. Our results showed that FxUD displayed inhibitory role in NLRP3 expression under uric acid stimulation. Subsequently, the protein level of ASC was reduced, leading to cleaved-caspase-1 down-regulation. To investigate the importance of NF-κB/NLRP3 signaling in inflammation reduction in tubular cells, we treated HK-2 cells with uric acid in the absence or presence of specific NF-kB inhibitor (NF-κB SN50) and NLRP3 inhibitor (MCC950), Western blot analysis was performed to detect the changes in the levels of key proteins. As shown in Figures 9D,E, SN50 and MCC950 reduced the levels of p-NF-κB and NLRP3 in HK-2 cells exposed to uric acid. More importantly, the protein levels were also restored significantly by FxUD treatment. These results collectively demonstrated that FxUD had a potential role in alleviating inflammation response by NF-κB/NLRP3 inhibition in UA-induced HK-2 cells.

### The Assessment of FxUD on Cardiovascular Safety

**FIGURE 10:**
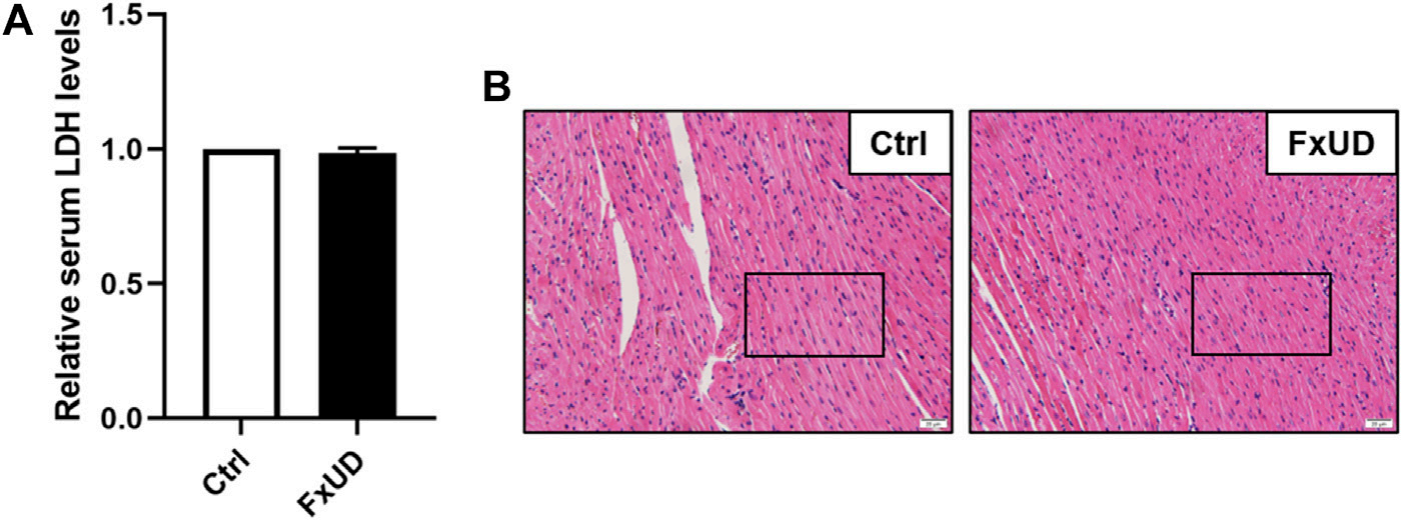
The effect of FxUD on cardiovascular safety. **(A)** The serum LDH levels. **(B)** Cardiotoxicity was assessed by HE staining to determine the effect on heart, no significant histological changes were noted. Magnification 200×, scale bar 20 µm. Values are expressed as the means ± SD (*n* = 4). *p < 0.05 vs. Ctrl.

Currently, drug-induced toxicity concluding nephrotoxicity, hepatotoxicity and cardiotoxicity is widespread. A major concern in drug development is toxicity to organs (Russell et al., 2017). Therefore, it is necessary to evaluate cardiotoxicity of the novel compound FxUD. Lactate dehydrogenase (LDH) is the commonly used indicator to assess the heart toxicity (Zhou et al., 2016). As shown in Figure 10A, serum LDH level indicated that FxUD did not exhibit cardiac toxicity compared with the control group. Meanwhile, we performed HE staining to determine the effect of FxUD on the hearts of mice (Figure 10B). Results revealed that the structure of myocardial cells was also normal, the muscle fibers were intact, and there was no inflammatory infiltration, myocardial fibrosis or myocardial necrosis. Therefore, only tentative conclusions could be drawn that FxUD was not cytotoxic on the heart and may serve as candidate drug for further investigation.

## Discussion

In recent years, the prevalence of hyperuricemia has increased, and more and more young people are suffering from this disease (Yong et al., 2018). Febuxostat is more effective and safer than allopurinol, although this drug has not been on the market for a long time. However, concerns about the cardiovascular safety of febuxostat cause reconsideration of the application of febuxostat (Bardin and Richette, 2019). Therefore, the search for natural products or compounds of high efficacy and safety has important significance. In our study, targeting of the therapy of hyperuricemia, we had synthesized a new compound named FxUD on the basis of febuxostat. Nephropathy induced by hyperuricemia is a common complication of hyperuricemia. Although the pathogenesis of renal complications caused by hyperuricemia is unclear, hyperuricemia has been considered as an independent risk factor for renal disease (Balakumar et al., 2020) and has been put forward to be usually recognized as a marker of renal abnormalities (Kang et al., 2002). Recent evidences clearly suggest that the pathogenic role of high uric acid in renal dysfunction involves a variety of pathological and molecular mechanisms (Kang, 2018). Thus, this study is meaningful in that it investigates the uric acid-lowering effects of the new compound and delineates the possible molecular mechanisms by which it ameliorates renal damage induced by hyperuricemia.

Serum urate level is the representative hallmark of hyperuricemia (Ichida et al., 2012). Urate is mainly synthesized in the liver, and nearly two-thirds of urate is excreted through the kidney daily (Vitart et al., 2008). Therefore, underexcretion of uric acid can cause kidneys damage. Cr and BUN levels are the most important biochemical makers to detect abnormal renal function (Wang et al., 2018). The present study showed that FxUD dose-dependently reduced the levels of uric acid, Cr and BUN in serum, and alleviated potassium oxonate induced hyperuricemia. Simultaneously, renal histological examinations also indicated that potassium oxonate induced lesions in kidney tissues to some extent, which could be obviously reversed by FxUD treatment, which further exhibited the involvement of hyperuricemia associated with inflammatory mechanisms. In fact, increasing evidence supports the conclusion that inflammation is the major mechanism for renal injury in rodents and patients with hyperuricemia (Malik et al., 2016).

In general, the levels of uric acid in the serum are regulated by a balance between uric acid production and excretion. The higher xanthine oxidase (XOD) activity can lead to excessive synthesis of uric acid (Zhao et al., 2006). XOD is a critical enzyme involved in uric acid production, and as a result, inhibiting the activity of XOD may be a feasible and effective way of controlling hyperuricemia. Our results showed that treatment with FxUD could significantly suppress XOD levels in serum and the liver, indicating that one of the potential mechanisms of FxUD on lowering uric acid may be because of the inhibitory effect on XOD levels.

Inflammation is a vital mechanism in the occurrence and maintenance of kidney injury (You et al., 2019). We further examined the changes in the expression levels of common inflammatory cytokines in kidney tissues. The results confirmed that FxUD significant reversed the elevation of inflammatory cytokines in kidney, indicating that the nephroprotective effects of FxUD might be attributed to its anti-inflammatory effect.

The clearance of uric acid is achieved through the interaction between the reabsorption and secretion of uric acid in the kidney, and is related to a variety of transporters present in the kidney (Lee et al., 2019). Among them, URAT1 and GLUT9 are important transporters that mediate the reabsorption of uric acid and the primary targets in the development of novel anti-hyperuricemic drugs. The results revealed that oral administration of FxUD significantly decreased mRNA and protein levels of renal URAT1 and GLUT9. In addition, genome-wide association studies of serum uric acid showed that several transporters such as ABCG2 (Nakayama et al., 2011) and organic anion transporter 1 (OAT1) (Hediger et al., 2005) also play a significant role in uric acid excretion. Thus, the effects of FxUD on uric acid excretion via the kidney are worthy of further research.

It has been reported that the increase in IL-1β and IL-18 expressions is closely related to activation of the NLRP3 inflammasome pathway. Our above results also found that uric acid could induce release of IL-1β consistently with the previous studies (Nicholas et al., 2011). In addition, increasing evidence have demonstrated that oxidative stress can increase the production of oxidative enzyme and then induce an increase in ROS, thereby activate NLRP3 inflammasome signal pathway and up-regulate the expressions of pro-inflammatory cytokines (Sharma et al., 2018). The NLRP3 inflammasome complex embraces NLRP3, ASC adaptor and caspase-1. Our results showed that potassium oxonate could resulted in increased expression of NLRP3, ASC adaptor and cleaved-caspase-1 in experimental mice, and these alterations could be remarkably reversed by FxUD treatment suggesting that FxUD might alleviate kidney injury via regulating NLRP3 inflammasome signal pathway in hyperuricemic mice.

High uric acid level can stimulate NF-κB activation in primary renal proximal tubule cells (Han et al., 2007). NF-κB has long been regarded as a typical pro-inflammatory signaling pathway, mainly based on the activation of NF-κB induced by pro-inflammatory cytokines, and NF-κB acts as a master regulator of pro-inflammatory responses to modulate expressions of pro-inflammatory genes including cytokines, chemokines, and adhesion molecules, which has been fully researched elsewhere (Lawrence, 2009). During inflammatory reaction activation, increased AMPK phosphorylation enhances SIRT1 expression, and ultimately decreases NF-κB p65 acetylation and nuclear translocation. Less inflammatory cytokines release further attenuates NF-κB p65 acetylation and transport through lower NF-κB p65 inhibitory factor IκBα phosphorylation and degradation (Zhang et al., 2016). What’s more, NF-κB pathway signaling was involved in the KEGG enrichment analysis between the model and FxUD treat group. In order to clarify the specific involvement of this signal transduction mechanisms, we performed further experimental validation. In our study, the results showed that NF-κB p65 and IκBα were significantly activated in hyperuricemic mice induced by potassium oxonate, and FxUD was found to suppress renal NF-κB signaling and exerted its functions on reducing inflammation in kidney of hyperuricemic mice.

Moreover, the disturbance on NF-κB signaling and NLRP3 inflammasome was detected in uric acid-exposed HK-2 cells *in vitro*. Results showed that FxUD treatment was associated with reduced expressions of inflammatory cytokines and uric acid reabsorption transporters, and also demonstrated that FxUD had a potential role in suppressing inflammation response via NF-κB/NLRP3 activation in uric acid-induced cells. Finally, to assess the underlying influence of FxUD on cardiovascular and safety, we selected two common indications which were linked to cardiotoxicity, the results indicated that FxUD had no noticeable toxicity on the heart.

**FIGURE 11:**
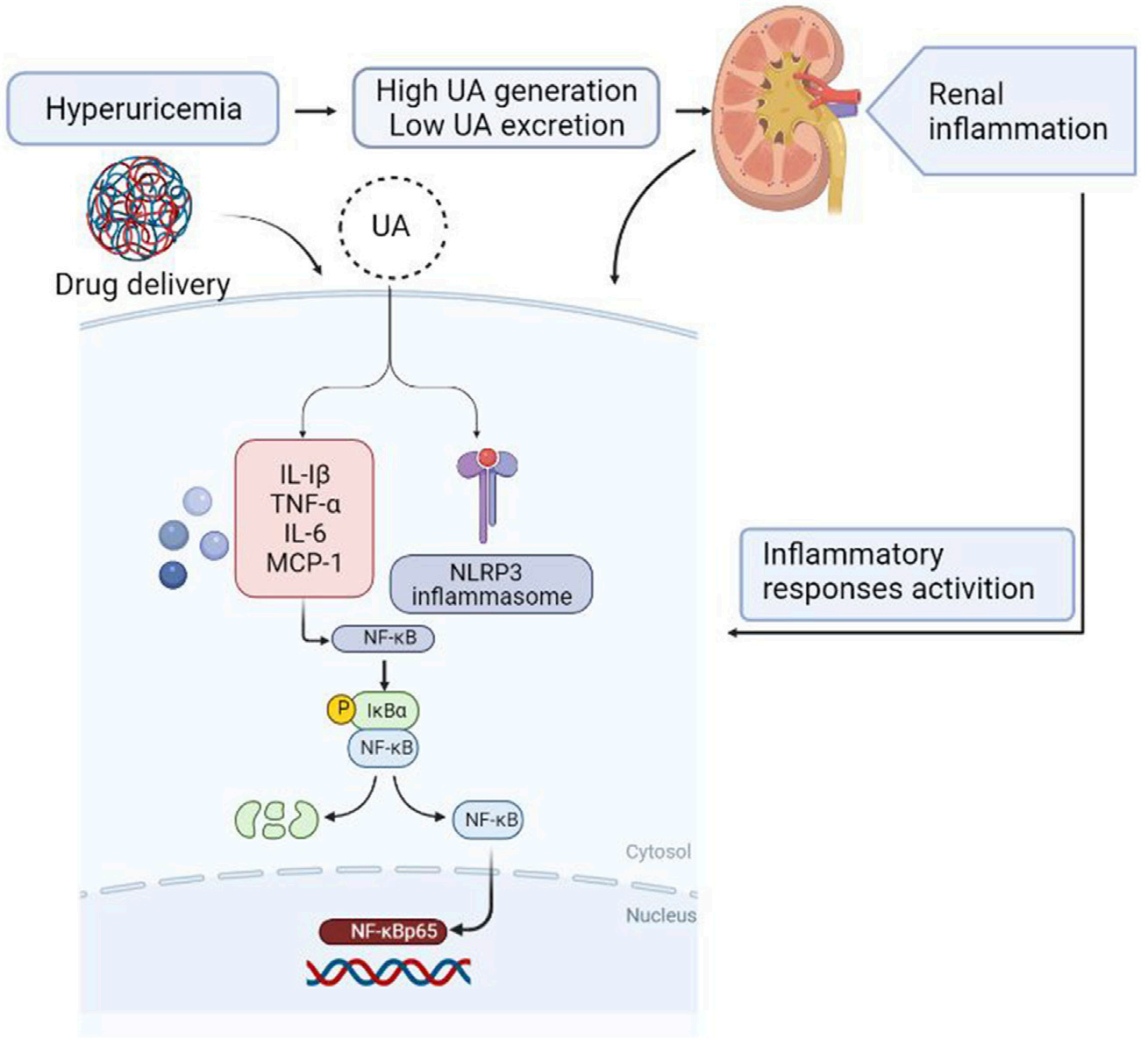
Diagram of the speculative mechanism how the drug affects inflammation induced by hyperuricemia in the kidney generated using BioRender.

In summary, this study shows that the anti-hyperuricemic and anti-inflammatory effects of FxUD in potassium oxonate-induced hyperuricemic mice and HK-2 cells exposed to uric acid. The mechanisms of its anti-hyperuricemic effect may be due to its significant inhibition against XOD, down-regulation of URAT1 and GLUT9. The molecular mechanisms involved in inflammatory response in the kidney under high serum uric acid level attribute to suppression of NF-κB signaling and NLRP3 inflammasome activation (Figure 11). Results from our research provide further insights for the development of effective and safe therapeutic candidates to combat hyperuricemia.

## Data Availability

The datasets analyzed in this article are publicly available. The RNA-seq data presented in the study are deposited in the public repository with GEO accession number GSE186871 or SRA accession number SRP343909. Requests to access further datasets should be directed to JW, wangjinhui@hrbmu.edu.cn.
